# Increased expression of lung TRPV1/TRPA1 in a cough model of bleomycin-induced pulmonary fibrosis in Guinea pigs

**DOI:** 10.1186/s12890-019-0792-z

**Published:** 2019-02-04

**Authors:** Yali Guo, Sun Ying, Xuehui Zhao, Jian Liu, Yuguang Wang

**Affiliations:** 1 Department of Respiratory Medicine, Beijing Hospital of Traditional Chinese Medicine, Capital Medical University, No. 23rd Art Museum Backstreet, Dongcheng District, Beijing, China; 2 Department of Immunology, School of Basic Medical Sciences, Capital Medical University, No. 10rd Xitoutiao, You’anmenwai street, Fengtai District, Beijing, China; 3 Department of Respiratory Medicine, Beijing Hospital of Traditional Chinese Medicine, Capital Medical University, No. 23rd Art Museum Backstreet, Dongcheng District, Beijing, China; 4 Department of Respiratory Medicine, Beijing Hospital of Traditional Chinese Medicine, Capital Medical University, No. 23rd Art Museum Backstreet, Dongcheng District, Beijing, China; 5 Department of Respiratory Medicine, Beijing Hospital of Traditional Chinese Medicine, Capital Medical University, No. 23rd Art Museum Backstreet, Dongcheng District, Beijing, 100010 China

**Keywords:** Cough, Idiopathic pulmonary fibrosis, Micro-CT, TRPV1/TRPA1

## Abstract

**Background:**

Chronic cough is a difficult-to-treat comorbidity of idiopathic pulmonary fibrosis (IPF), and significantly impacts on the quality of life of patients with IPF. Transient receptor potential (TRP) channel proteins may play an important role in chronic cough. However, expression of these proteins in lung of IPF is largely unknown.

**Methods:**

Guinea pig model of pulmonary fibrosis was established by single intratracheal delivery of bleomycin. Respiratory ungated micro-CT scans were performed on days 7, 14, 21 and 28 to assess progression of pulmonary fibrosis. Cough sensitivity to capsaicin was evaluated in conscious animals on days 13 and 27. Real-time PCR (qPCR) and immunohistochemistry were employed to measure expression of TRPV1 and TRPA1 in lung tissue.

**Results:**

Micro-CT showed that lung consolidation was detectable from day 7 distributing mainly in the middle and lower lung fields, which was significantly correlated to Ashcroft fibrosis score (r = 0.7993, *p* < 0.001). Cough sensitivity to capsaicin in bleomycin-treated animals was significantly increased on days 13 and 27. qPCR showed that expression of TRPV1 and TRPA1 was positively correlated each other and significantly upregulated in lung tissues of model group compared with that of controls, which was further supported by immunohistochemistry. Furthermore, immunoreactivity for TRPV1 and TRPA1 was negatively correlated with Ashcroft fibrosis score.

**Conclusion:**

Expression of TRPV1/TRPA1 was upregulated in the chronic cough related to bleomycin induced pulmonary fibrosis in guinea pigs, which provided new insights into the mechanism of IPF-associated cough hypersensitivity. Micro-CT is very helpful methodology to access pulmonary fibrosis progression in small animal models.

## Background

Idiopathic pulmonary fibrosis (IPF) is a chronic progressive fibrotic lung disease of unknown cause, with a median survival of 2~3 years after diagnosis [1]. Cough, breathlessness and fatigue are the most common symptoms of IPF [2]. It has been reported that up to 80% of patients with IPF have chronic cough [3], which severely affects IPF patients on various aspects of life, and even decreases quality of life [4]. On the other hand, cough severity might predict disease progression and severity in IPF [3]. Furthermore, cough is one of the most difficult-to-treat comorbidities of IPF, which is often refractory to conventional anti-tussive therapy [5].

Few studies have focused on the treatment and the mechanism of chronic chough related to IPF. Some small, single-centre trials have shown that low doses of prednisone, thalidomide and interferon-α could ameliorate cough [6–8]. Considering side-effects and commercial burden, however, these drugs have not been recommended for IPF cough treatment. The pathophysiology of chronic cough in IPF is still unknown [9]. Transient receptor potential (TRP) channels in the respiratory system are sensitive to thermal and chemical stimulation, which possibly play important roles in the mechanisms of chronic cough [10], particularly, TRP channel subfamily vanilloid member 1 (TRPV1) and TRP channel subfamily A member 1 (TRPA1). TRPV1 and/or TRPA1 can be sensitised indirectly by a range of inflammatory mediators, including bradykinin, nerve growth factor and so on. It has been shown that cough sensitivity to chemical stimuli (inhaled capsaicin or substance P) increased in patients with IPF and had higher levels of neurotrophins in the sputum [11].

Thus, it is necessary to establish an animal model of cough related to IPF for further understanding pathophysiology and exploring new treatments for the disease. Herein, we hypothesized that the cough reflex increases in a model of bleomycin-induced pulmonary fibrosis in guinea pigs, which is associated with up-expression of TRPV1 and TRPA1. In addition, we evaluated in vivo micro-computed tomography (CT) as a non-invasive tool to assess progression of pulmonary fibrosis in guinea pigs.

## Methods

### Experimental materials

Specific pathogen-free male Dunkin–Hartley guinea pigs (250~300 g) were purchased from Vital River Laboratories (Beijing, China). Bleomycin (BLM) was purchased from Hisun-Pfizer Pharmaceuticals (Zhejiang, China). Capsaicin was purchased from Sigma Aldrich (Seattle, USA). PureLink™ RNA Mini Kit was purchased from Thermo Fisher Scientific (Carlsbad, USA), One-Step PrimeScript™ RT-PCR Kit from TaKaRa (Dalian, China). Primers were synthesized by Sangon (Shanghai, China). TRPV1 primary rabbit antibody was purchased from Abcam (Cambridge, MA, USA) and TRPA1 primary rabbit antibody from Novus-Bio (Littleton, USA). The poly-HRP anti-rabbit IgG secondary antibody and DAB solution were purchased from ZSGB-Bio (Beijing, China). Hydroxyproline assay kit was purchased from Jiancheng Bioengineering Institute (Jiangsu, China).

### Animals

Guinea pigs were housed in a specific pathogen-free facility located in the Beijing Institute of Microbiology and Epidemiology, Beijing, China. Animals were housed in filtered ventilated cages in a room with controlled temperature (20~24 °C), humidity (40~60%), air cycles (10~20 renovations/h) and a 12 h light/12 h dark cycle conditions. Bleomycin was dissolved in phosphate buffer saline (PBS) (pH 7.4) and immediately used after preparation.

### Model of pulmonary fibrosis

Forty guinea pigs were randomly divided into three groups (12~14 per group): PBS group, BLM 14d group, BLM 28d group. Briefly, animals were anesthetized with 1% pentobarbital sodium (30 mg/kg, i.p.). A midline incision was made in the neck 1~2 cm upon the suprasternal fossae and the trachea was exposed by blunt dissection. Bleomycin (8 IU/kg, dissolved into 200 μL PBS) was single intratracheally administrated to animals of BLM groups. Administration of equivalent volume of PBS was used as the controls to PBS group. The skin and tissue in front of the trachea were sutured simply after operation. The signs of the disease and bodyweight were recorded daily till days 28 post-administration. Respiratory ungated micro-CT was used to assess the model of pulmonary fibrosis on days 7, 14, 21 and 28 after bleomycin administration dynamically without sacrificing the experimental animals. We assessed the cough reflex and expression of TRPs after 13~14d and 27~28d of BLM administration. The same indexes of guinea pigs in PBS group were measured on days 27~28. At the designated endpoint, animals were anesthetized with 1% pentobarbital sodium (30 mg/kg, i.p.) after evaluating of the cough reflex and micro-CT. Half of the animals were transcardially perfused with 4% paraformaldehyde in PBS (pH 7.4; 200 mL), and the lungs were removed and fixed in 4% paraformaldehyde. The remaining animals were sacrificed by femoral artery bleeding, and lung tissues were harvested and preserved in liquid nitrogen immediately. All experimental procedures involving animals were performed according to protocols approved by the Animal Experiment Committee of Laboratory Animal Center, Academy of Military Medical Sciences (IACUC-13-2016-001).

### Cough reflex

On days 13 and 27 after bleomycin administration, cough reflex was evaluated in conscious unrestrained guinea pigs as described previously [12]. Briefly, animals were placed in a transparent cubical container (volume, 3000 mL) which was continuously filled with room air using an air compressor pump. Capsaicin (15.25 mg) was dissolved in 0.5 mL pure ethanol and 0.5 mL Tween 80 and then further dissolved in 4 mL physiological saline solution to yield a 0.01 M stock solution, and further diluted with saline to obtain a final concentration (75 μM) before nebulization. Capsaicin solution was delivered to the chamber by aerosol with a Compressor Nebulizer (OMRON, Dalian, China) (producing particles in size of < 5 μm) for 5 min, followed by a further 5 min observation period. Coughs were counted during 10 min by a trained observer and recognized by characteristic animal posture (characteristic opening of the mouth associated with cough, splaying of the front feet and forward stretching of the neck) and sound [13].

### Micro-CT scans of animals

A CT scanner (Siemens Medical Solutions, Knoxville, TN, USA) was used for data-acquisition in prone position under isoflurane inhalation anaesthesia (tube voltage 80 kV, tube current 500 μA, effective pixel size 220 μM) on days 7, 14, 21 and 28 respectively after bleomycin administration. Scanning took approximately 6 min without respiratory gating. Respiratory monitoring was performed using a pressure transducer pad under the animal’s chest. The consecutive CT data were imported to Materialise’s interactive medical image control system (Mimics) (Materialise, Leuven, Belgium). Images were reconstructed and assessed at a constant window width/window level 2000/100). After selecting specific layers of images, the aerated lung areas and the total lung tissue areas were quantified using Image-Pro Plus 6.0 software (Media Cybernetics Corporation, USA). The lung consolidation values were represented by the ratio of consolidated lung area to total lung tissue area (calculated as (1- aerated lung area/total lung tissue area) × 100%). The lung consolidation values of the whole lung were represented by the mean value of each layer.

The chest micro-CT images were divided into six parts by five specific layers from rostral to caudal side. Images of specific layers in the coronal plane were defined as follows: layer 1, the level of carina; layer 2, the level of left upper lobe bronchus bifurcation; layer 3, the level where the inferior vena cava just appeared; layer 4, the level of the apical where the apex was minimal, while the top of the diaphragm not appeared yet; and layer 5, the level where the inferior vena cava just disappeared.

### Hydroxyproline assay

Collagen contents in the lungs were measured using a commercial hydroxyproline assay kit according to the manufacturer’ instructions.

### Lung histology and assessment of pulmonary fibrosis

The lungs were fixed in 4% paraformaldehyde for 24 h at 4°C. After fixation, the lungs were embedded with paraffin wax. Sections were serially cut at a thickness of 5 μm then stained using haematoxylin and eosin (H&E) and Masson’s trichrome to assess the degree of fibrosis after dewaxing and rehydration. The degree of lung fibrosis was determined according to the method described by Ashcroft et al. [14], employing a numerical scaling system in lung samples ranging from 0 (normal lung) to 8 (total fibrous obliteration of the field). The mean degree of pulmonary fibrosis was calculated from individual scores of six visual fields per guinea pig randomly.

### Gene expression

To detect expression of TRPV1 and TRPA1, total RNA was extracted from the frozen lung tissues with the PureLink™ RNA Mini Kit according to the manufacturer’s instructions and eluted in 50 μL of RNase-free water. RNA was quantified by a NanoDrop 1000 Spectrophotometer (Thermo Fisher Scientific, Carlsbad, USA) and diluted to 500 ng/μL. Real-time PCR was carried out in a total volume of 20 μL with the One-Step PrimeScript™ RT-PCR Kit and the LightCycler®480 system (Roche Diagnostics, Mannheim, Germany) according to the manufacturers’ instructions. Relative quantification of gene expression was assessed by the 2^-ΔΔt^ method [15] and β-actin served as an internal reference gene. Furthermore, to evaluate expression of inflammatory mediators and neutrophins, we also measured mRNA expression of interleukin-8 (IL-8) and brain-derived neurotrophic factor (BDNF) in the lung tissues. Genus-specific primers were designed using NCBI/Primer-BLAST. The sequences and lengths of the amplified fragments were as follows (Table 1).

**Table 1:** The sequences and lengths of the genus-specific primers

β-actin (331 bp)	Forward 5’-CCATCTACGAGGGCTACG-3’
	Reverse 5’-ATGCCACAGGATTCCATAC-3’
TRPV1 (284 bp)	Forward5’-CAGAGAGCCATCACCATCCT-3’
	Reverse 5’-GGGACCAGGGCAAAGTTC-3’
TRPA1 (148 bp)	Forward 5’-ACAATGATGGCTGCACTCCT-3’
	Reverse 5’-ATACGCCCATAACTGGCTGC-3’
BDNF (147 bp)	Forward 5’-TAAAAAGACGGCCGTGGACA-3’
	Reverse 5’-TTGTCTATGCCTCTGCAGCC-3’
IL-8(81 bp)	Forward5’-CCTTGGATTCCCCTTTATTCCT-3’
	Reverse 5’-CGTATGTCCCCATGACATTGTG-3’

### Immunohistochemistry

Immunoreactivity for TRPV1 and TRPA1 in lung tissues was measured using immunohistochemistry. Briefly, after dewaxing and rehydrating, sections (5 μm thickness) were incubated with 3% H_2_O_2_ for blocking endogenous peroxidase, and then treated with EDTA (pH 8.0) for 3 min in high pressure cooker for antigen retrieve. To reduce nonspecific binding, sections were incubated with 10% normal goat serum for 20 min at 37 °C. Sections were then incubated with primary rabbit antibodies against TRPV1 (1:2200) and TRPA1 (1:3500) at 4 °C overnight. After washing for 3 times with PBS, sections were incubated with poly-HRP anti-rabbit IgG secondary antibody (1:1000) for 20 min. Positive signals were developed using DAB solution. The stained tissue sections were counterstained with hematoxylin. Immunoreactivity for TRPV1 and TRPA1 was visualized under an Olympus DP70 microscope (Olympus Corporation, Tokyo, Japan) and scanned using NanozoomerS210 whole slides scanner (Hamamatsu Photonics, Hamamatsu City, Japan). Six visual fields were chosen randomly per guinea pig, and images were analysed with Image-Pro Plus 6.0 software.

### Statistical analysis

All data are expressed as the mean ± SEM. Statistical computations were performed using the statistical program GraphPad Prism 5.0. Differences between groups of animals were compared using the two-tailed, unpaired Student’s *t* test. Correlation of continuous variables was calculated using a Pearson correlation. Statistical significance was defined as *P* < 0.05.

## Results

### Signs of disease and bodyweight

**Fig. 1:**
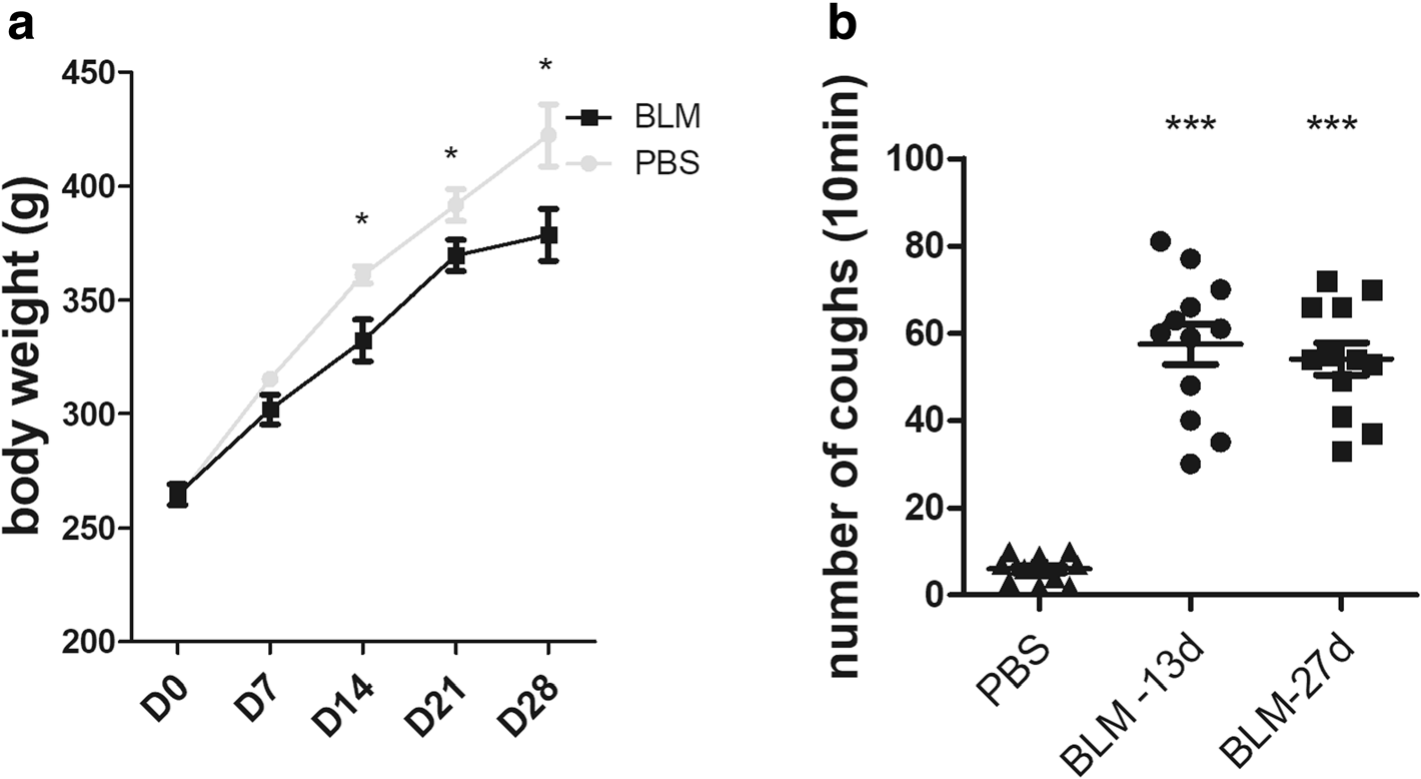
**a** Changes of body weight on days 0, 7, 14, 21 and 28. **b** Altered cough reflex to capsaicin (75 μM) after bleomycin intratracheal administration in guinea pigs on days 13 and 27. (*n* = 12~14 animals per group). *p < 0.05, ***p < 0.001, vs. PBS group

The guinea pigs in the PBS group had flexible activities, bright and clean fur, normally eating and drinking, stably gaining in weight during the experiment. In contrast, guinea pigs in the BLM group had shortness of breath, reduced food and water intake, less movement, fluffy fur, cyanosis in claws and mouth, poor mental response, which were most obvious during 1 to 5 days after modelling. The gain in weight was significantly slower than that of PBS group (Fig. 1a, *p* < 0.05 from day 14).

### Measurement of cough reflex

To investigate whether the guinea pig model of bleomycin-induced pulmonary fibrosis is suitable to study pulmonary fibrosis cough, the cough reflex was recorded throughout experiments. The results revealed that the number of coughs induced by aerosolized capsaicin (75 μM) was significantly increased on days 13 and 27 in the BLM-treated group compared with the PBS-treated group (Fig. 1b, mean ± SEM: 57.5 ± 4.66 and 54.17 ± 3.68 vs. 6.0 ± 0.81, *p* < 0.001, respectively).

### In vivo micro-CT imaging of lungs after BLM-treated

**Fig. 2:**
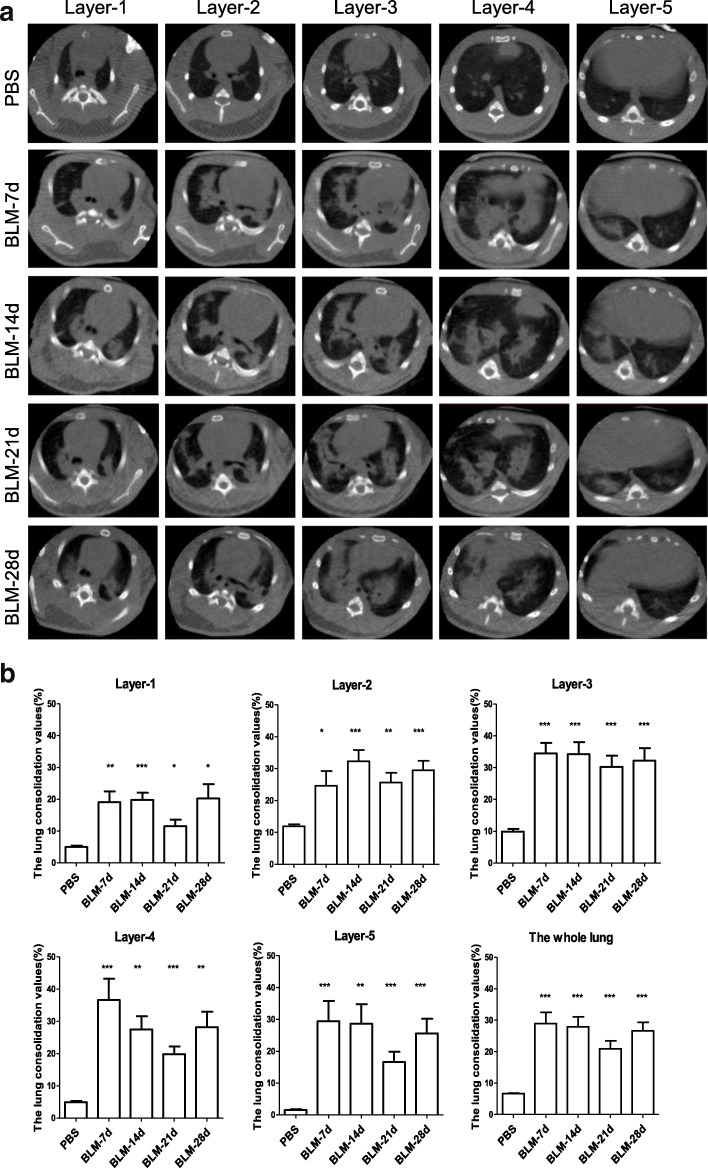
Respiratory ungated micro-CT on days 7, 14, 21 and 28 at five different layers after intratracheal bleomycin administration. **a** Typical micro-CT images of a guinea pig at different time points. **b** The values of the lung consolidation at the different layers with corresponding time points (*n* = 6~7). *p < 0.05, **p < 0.01, *** *p* < 0.001, vs. PBS group

Micro-CT imaging revealed that there was strong consolidation in the lungs of animals after intratracheal bleomycin administration. The consolidation was detectable as early as on day 7, particularly on days 14 and 28 post-administration (Fig. 2a). Quantitative analysis showed that the values of the lung consolidation were significantly higher at each layer of the BLM group than that of the PBS group (Fig. 2b). Pathological changes were focally distributed in the middle and lower lung fields (layer 2 to 4), along the peripheral bronchovascular bundle.

### The degree of pulmonary fibrosis after BLM-treated

**Fig. 3:**
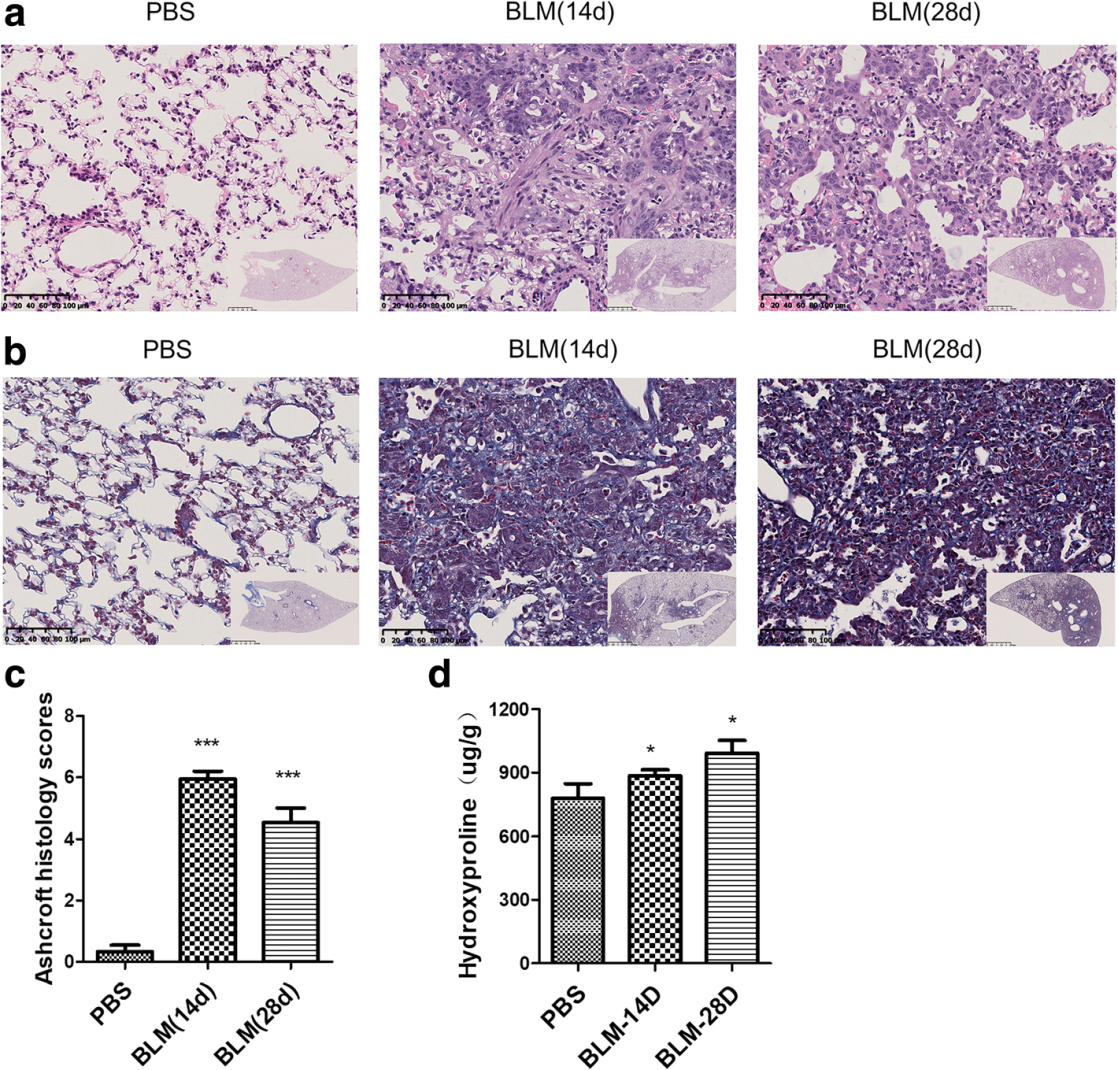
Histopathological changes of lung tissue in guinea pigs on days 14 and 28 after BLM administration. **a** H&E staining shows pulmonary inflammation, fibrosis, and integrity of the structure (original magnification× 20). **b** Masson’s staining shows deposition of collagens in lung tissues (original magnification× 20). **c** and **d** Degree of pulmonary fibrosis was evaluated by Ashcroft fibrotic scores and hydroxyproline (*n* = 6~7). *p < 0.05, ***p < 0.001 vs. PBS group

The micro-CT in vivo showed that the pathological changes were mainly distributed in the middle and lower lung fields. Accordingly, lung tissues at the middle and lower fields were embedded for pathology. Histological staining with H&E and Masson trichrome showed that there were obvious infiltrating inflammatory cells, fibrosis, thickening of alveolar walls, and distortion of the architecture of the pulmonary parenchyma. These changes were obviously around bronchovascular bundle areas in BLM-treated group on days 14 and 28 (Fig. 3a and b), which were consistent with what were observed from micro-CT imaging in vivo. Correspondingly, Ashcroft fibrotic scores showed that there was significant difference in the severity of fibrosis between BLM and PBS group (Fig. 3c, *p* < 0.001).

Pulmonary fibrosis is characterized by collagen accumulation, while hydroxyproline is a major component of the collagen and can be used as an indicator to determine the amount of collagen. Hydroxyproline measurement showed that the concentrations of hydroxyproline were elevated at day 14 and significantly increased at day 28 compared with that of PBS group (Fig. 3d, *p* < 0.05).

### Immunohistochemistry of TRPs in the lung

**Fig. 4:**
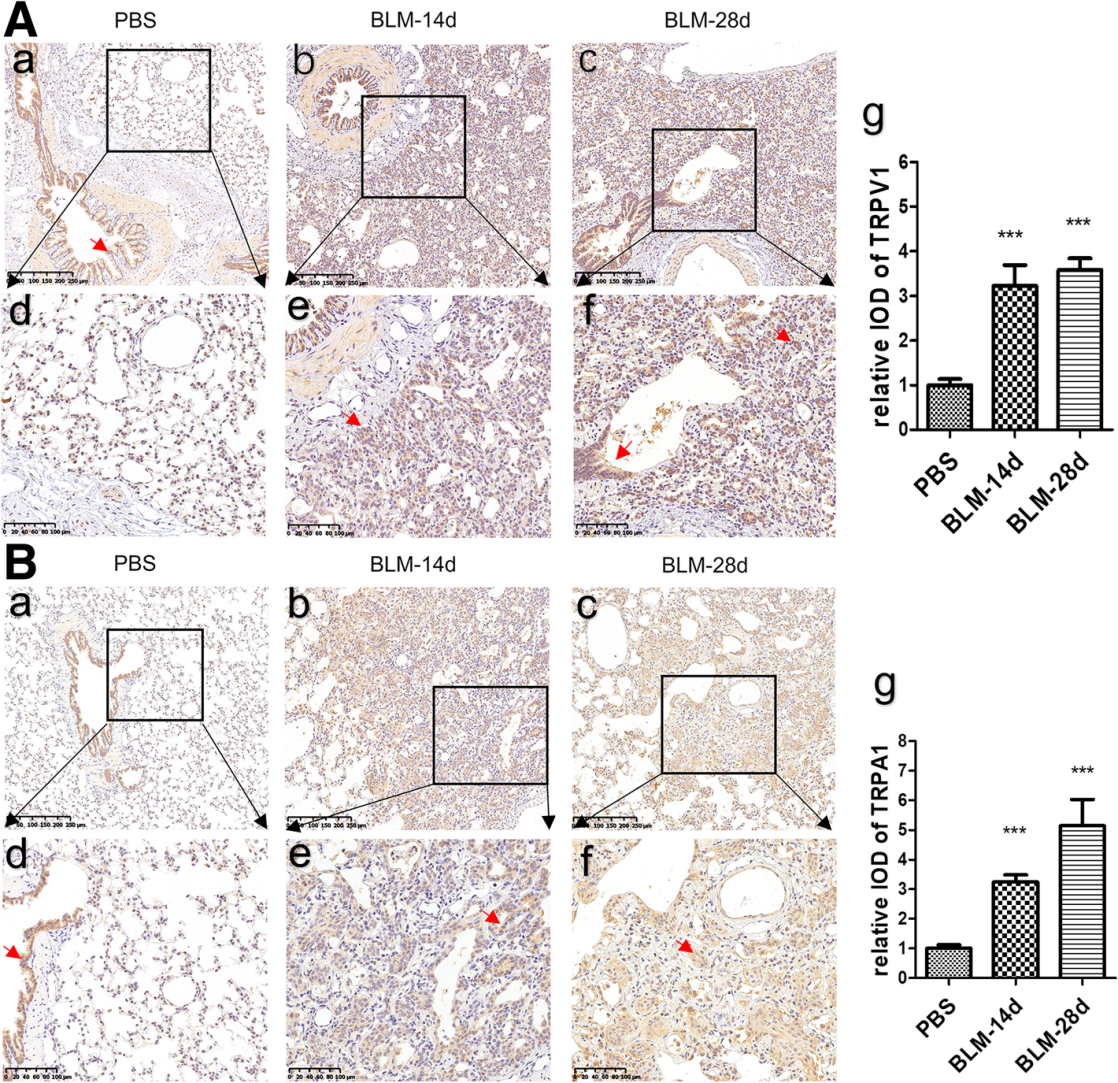
Immunoreactivity for TRPV1 and TRPA1 in lung sections of bleomycin-induced pulmonary fibrosis in guinea pigs. **A** (a, b, c) and **B** (a, b, c): Immunohistochemical stain of TRPV1(**A**) and TRPA1 (**B**) proteins (original magnification × 20). **A** (d, e, f) and **B** (d, e, f): Partial magnification(original magnification × 40) of **A** (a, b, c) and **B** (a, b, c). **A** (g) and **B** (g) show IOD value of immunoreactivity for TRPV1 and TRPA1 analyzed with IPP software (n = 6 per group). ***p < 0.001 vs. PBS group. Red arrows point to the positive expression area

The TRP channel proteins were initially thought to be localized to a subset of sensory nerve fibres in the respiratory tract and were sensitive to various noxious stimuli and temperature. Later studies found that they were also expressed in several respiratory nonneuronal cells. It has been shown that the neuronal and nonneuronal TRPs contribute significantly to rhinitis, asthma, chronic obstructive pulmonary disease and chronic cough [10]. Immunostaining showed that immunoreactivity for TRPV1 and TRPA1 was mainly located in the airway mucosal epithelial cells, but hardly in the lung parenchyma of the PBS group (Fig. 4a and b). In contrast, in the BLM group immunoreactivity for these TRPs was markedly located on respiratory bronchiolar epithelial cells and hyperplastic alveolar epithelial cells in the lung parenchyma, but not in areas with large amounts of collagen fibres (Fig. 4a and b). Semi-quantitative analysis showed that immunoreactivity for TRPV1 and TRPA1 was significantly increased in BLM challenged animals compared with that of PBS controls on days 14 and 28 (Fig. 4a and b, *p* < 0.01 respectively).

### Gene expression of TRPs and cytokines in lung tissues

**Fig. 5:**
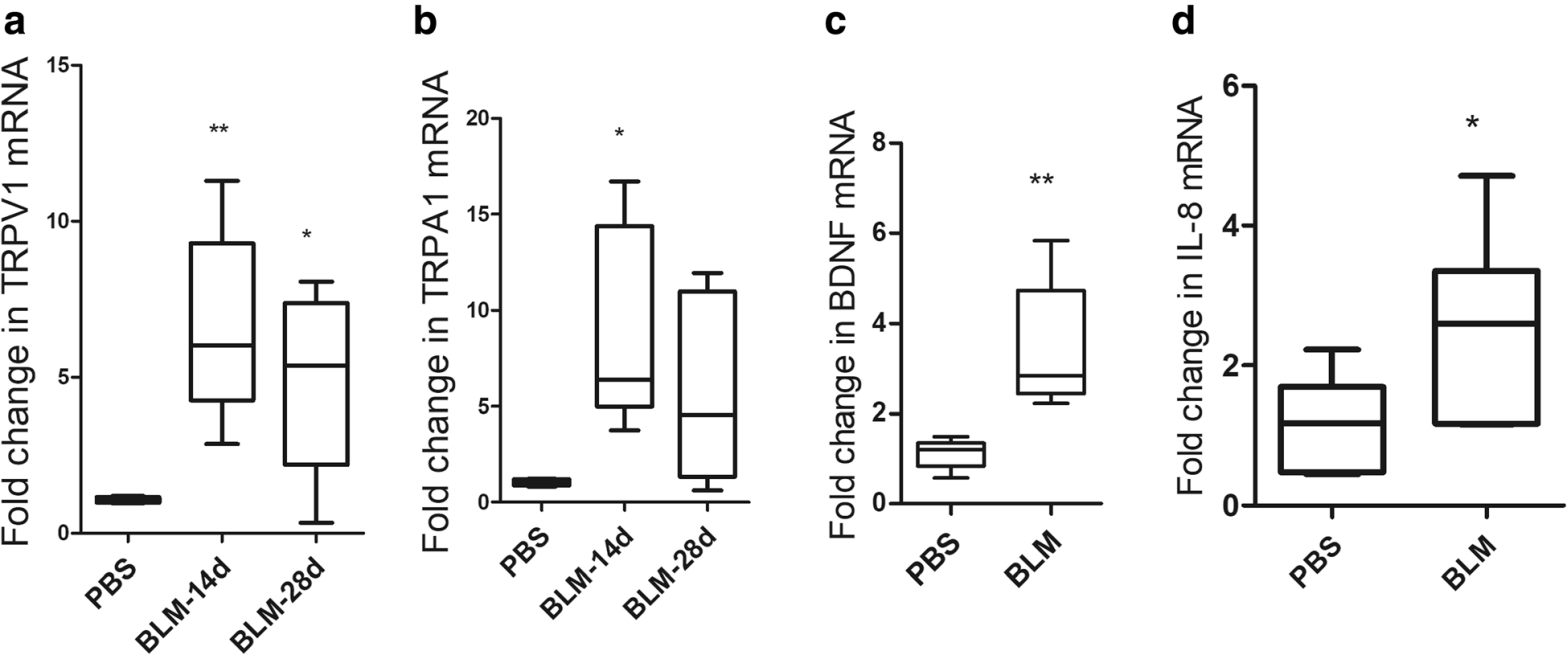
The mRNA expression of TRP channels and cytokines. Fold change in TRPV1 (**a**) and TRPA1 (**b**) mRNA expression at 14 and 28 days after treatment with bleomycin. BDNF (**c**) and IL-8 (**d**) mRNA expression at 28 days (*n* = 5~7 per group). **p < 0.01 and *p < 0.05, vs. PBS group

Real-time PCR showed that TRPV1 mRNA expression was significantly upregulated on days 14 (Fig. 5a, *p* < 0.01) and 28 (Fig. 5a, *p* < 0.05). Expression of TRPA1 mRNA was higher on days 14 compared with that of the control (Fig. 5b, *p* < 0.05). In the meantime, BDNF and IL-8 mRNA levels were also upregulated at days 28 (Fig. 5c and d, *p* < 0.05 respectively).

### Correlation analysis

**Fig. 6:**
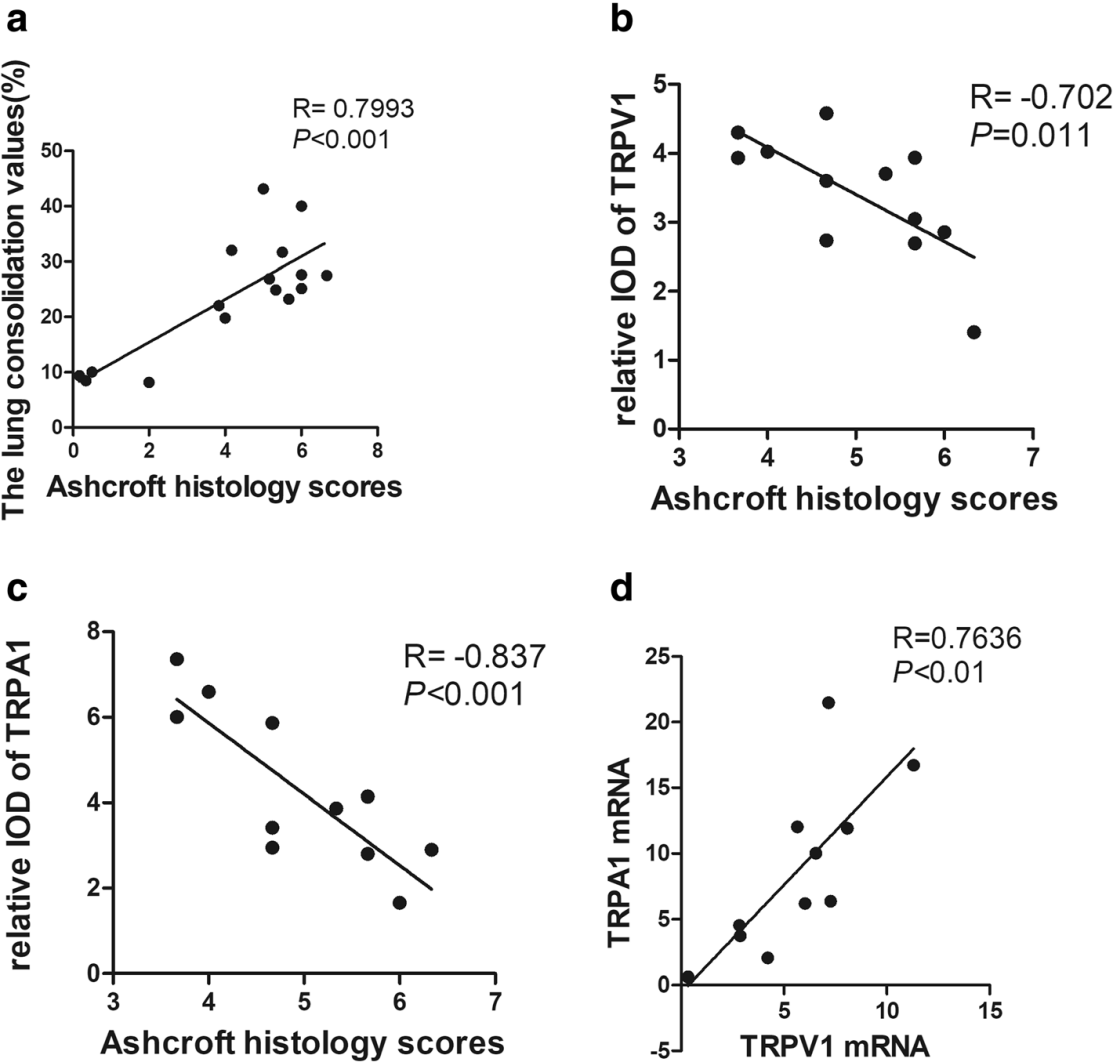
Correlation analysis. **a** Ashcroft fibrotic scores with the values of lung consolidation. **b** and **c**: Ashcroft fibrotic scores with immunoreactivity for TRPV1 (**b**) and TRPA1 (**c**) in lung sections. **d** Correlation of relative expression of TRPV1 mRNA with relative expression of TRPA1 mRNA of the same lung tissues

In the present study, we also evaluated micro-CT as a non-invasive method to access the lung lesions after intratracheal administration of bleomycin. We first analysed the correlation of histological grading of pulmonary fibrosis with the lung consolidation values in the middle and lower lung fields (layer 2 to 4). Results showed that there was a significantly positive correlation between Ashcroft fibrotic score and the values of lung consolidation (Fig. 6a, r = 0.7993, *p* < 0.001). Then we compared the Ashcroft fibrotic scores with expression of TRPV1 and TRPA1. Surprisingly, immunoreactivity for TRPV1 and TRPA1 negatively correlated with Ashcroft fibrotic score respectively (Fig. 6b and Fig. 6c, r = − 0.702, *p* = 0.011, r = − 0.837, *p* < 0.001). In addition, expression of TRPA1 positively correlated with TRPV1 at mRNA level in lung tissues (Fig. 6d, r = 0.7636, *p* < 0.01).

## Discussion

In our best knowledge, there is a lack of animal model currently available to fully recapitulate the progressive nature of IPF or the histological changes of usual interstitial pneumonia. Mice and rats are the most animals used for establishing models of pulmonary fibrosis induced by intratracheal administration of bleomycin. In these models, inflammatory and fibrotic reactions occur within 1~2 weeks, companying with increases in expression of interleukin-1(IL-1), tumor necrosis factor-α (TNF-α), interleukin-6 (IL-6), interferon-γ (INF-γ ), TGF-β, fibronectin and procollagen-1 [16]. These animals, however, can’t produce a cough that resembles the reflex seen in humans. In contrast, the guinea pig has similarity in response to both citric acid and capsaicin in humans [17], while coughing could be blocked by anaesthesia [18]. In this case, conscious guinea pigs become the most useful animal model for experimental studies of chemically induced cough [13]. In the present study, we established a model of cough hypersensitivity induced by a single intratracheal administration of bleomycin in conscious guinea pig. Obviously, this model was able to show cough hypersensitivity related to bleomycin induced pulmonary fibrosis. Although both citric acid and capsaicin can induce cough in a dose-dependent and reproducible manner to measure cough sensitivity, however, inhalation cough challenge using capsaicin is much safer compared with that of citric acid [13]. Our data showed that the guinea pigs with pulmonary fibrosis were much more sensitive to inhaled capsaicin than that of the control animals.

On the other hand, although histological and biochemical methods are common technologies to assess the degree of pulmonary fibrosis in small laboratory animals, performing these methods requires sacrificing the experimental animals after certain periods. In humans, non-invasive diagnostic tools such as chest CT scans and pulmonary function tests are normally used to assess the severity of pulmonary fibrosis. It has been shown that respiratory ungated micro-CT imaging in vivo has highly significant correlation with histological changes based on Ashcroft score, and offers a non-invasive tool to evaluate pulmonary fibrosis progression in mice [19]. Our data showed that respiratory ungated micro-CT in vivo revealed easily detectable consolidation from day 7, with ground-glass opacity around the consolidation. As time went on, edge of consolidation became clear gradually. In the meanwhile, the micro-CT data were further confirmed by the correlation between Ashcroft fibrotic score and the values of lung consolidation through micro-CT, supporting the idea that ungated micro-CT is able to reflect the real status of lungs during the pulmonary fibrotic process.

The mechanism of chronic cough related to IPF is still unclear. The basic physiological cough reflex pathways start from activation of vagal afferent fibres presented in the airways [20, 21]. The cell bodies of which mostly originate from the nodose and jugular ganglia while around 1% from the thoracic dorsal root ganglia (DRG) [22]. C fibres and Aδ nociceptors are the two fibre types which are more chemosensitive. Aδ nociceptors originate in the jugular ganglia and can be activated by TRPV1 agonists such as capsaicin, but their contribution to the cough reflex is still not clear. C fibres make up the majority of chemosensitive nerve fibres and can be activated by agonists of the TRPV1 (e.g. capsaicin) and TRPA1 (e.g. allylisothiocyanate, acrolein and cinnamaldehyde). They originate in both the nodose and jugular ganglia. In guinea pigs, TRPV1 is functionally expressed on airway jugular neurons while less on the nodose neurons [23]. Neurotrophins (including nerve growth factor (NGF), BDNF, neurotrophin-3(NT-3), NT-4) and inflammatory mediators (such as IL-6, TNF- α), could cause TRPV1 translocation to the cell membrane or are able to regulate the excitability of TRPV1/TRPA1 [24, 25]. For example, the nodose ganglia cells become much more responsive to capsaicin after exposure of BDNF or ovalbumin to the trachea of the guinea pig, suggesting the existence of neuroplasticity in the respiratory tract [26, 27]. TRPA1 is also a molecular target of oxidative stress [28]. Those may explain the increased cough sensitivity under disease condition. In addition, TRPV1/TRPA1 activation produces the release of IL-8 [29, 30]. In addition, BDNF and its high affinity receptor TrkB, but not NGF, NT-3 and their respective high affinity receptors TrkA and TrkC, were found strongly stained in the fibroblastic foci in IPF patients [31]. Therefore, it is reasonable to speculate that inflammatory response, oxidative stress, fibrosis in the process of pulmonary fibrosis may induce remodelling of sensory afferent fibres and neurogenic inflammation, upregulate the expression of TRP ion channels and increase cough hypersensitivity. Our data verified that the expression of TRPV1 and TRPA1 in lung tissues of the bleomycin-treated animals was significantly upregulated at both mRNA and protein levels. These findings are inconsistent with a previous study, in which the pulmonary mRNA expression of TRPV1, TRPA1 was unchanged [32]. One potential explanation might be due to the different efficiency of primers and the method of modelling used. In the present study, expression of mRNA encoding BDNF was also proved to increase in the lung tissues of bleomycin-treated animals. Furthermore, qPCR data also showed that expression of IL-8 was upregulated, which was possibly associated with the upregulation of TRPs.

Previous studies demonstrate that TRPs proteins mainly localize in the sensory network. In addition, a variety of human primary lung cells, such as bronchial epithelial cells, bronchial smooth muscle cells, fibroblast, also can express TRP channels proteins [10]. In the present study, immunoreactivity for TRPV1and TRPA1 mainly located in bronchiolar epithelial cells, hyperplastic alveolar epithelial cells and fibroblast, but rarely in areas with large amounts of collagen fibres. These features possibly explain why the TRPV1 and TRPA1 expression were negatively correlated with Ashcroft fibrotic scores. However, we did not find any correlation between cough numbers and expression of TRPV1 and TRPA1 (data not shown). Reasons may be listed as follows. Firstly, intratracheal instillation of bleomycin causes bronchiocentric-accentuated areas of fibrosis [33], while local pathological changes are difficult to reflect the overall situation. Secondly, many factors may contribute to the occurrence of cough related to pulmonary fibrosis, such as inflammatory mediators, neuropeptides, ROS and mechanical traction forces of fibrosis [9, 34].

## Conclusions

In summary, we established a guinea pig model with enhanced cough sensitivity related to bleomycin-induced pulmonary fibrosis and brought in micro-CT as a non-invasive assessment of pulmonary fibrosis. Upregulation of TRPV1 and TRPA1 in the lung tissues of the animal models suggest that the increased sensory unmyelinated C fibres and neurogenic inflammation may play a role in cough hypersensitivity of IPF.

## Abbreviations

BDNF: Brain-derived neurotrophic factor
BLM: Bleomycin
CT: Computed tomography
H&E: Haematoxylin and eosin
IL-8: Interleukin-8
IPF: Idiopathic pulmonary fibrosis
NGF: Nerve growth factor
qPCR: Real-time PCR
TNF-α: Tumor necrosis factor-α
TRP: Transient receptor potential
TRPA1: TRP channel subfamily A member 1
TRPV1: TRP channel subfamily vanilloid member 1
